# Tamoxifen and the Rafoxifene analog LY117018: their effects on arachidonic acid release from cells in culture and on prostaglandin I_2 _production by rat liver cells

**DOI:** 10.1186/1471-2407-4-49

**Published:** 2004-08-13

**Authors:** Lawrence Levine

**Affiliations:** 1 Department of Biochemistry, Brandeis University Waltham, MA 02454, USA

## Abstract

**Background:**

Tamoxifen is being used successfully to treat breast cancer. However, tamoxifen also increases the risk of developing endometrial cancer in postmenopausal women. Raloxifene also decreases breast cancer in women at high risk and may have a lower risk at developing cancer of the uterus. Tamoxifen has been shown to stimulate arachidonic acid release from rat liver cells. I have postulated that arachidonic acid release from cells may be associated with cancer chemoprevention.

**Methods:**

Rat liver, rat glial, human colon carcinoma and human breast carcinoma cells were labelled with [^3^H] arachidonic acid. The release of the radiolabel from these cells during incubation with tamoxifen and the raloxifene analog LY117018 was measured. The prostaglandin I_2 _produced during incubation of the rat liver cells with μM concentrations of tamoxifen and the raloxifene analog was quantitatively estimated.

**Results:**

Tamoxifen is about 5 times more effective than LY117018 at releasing arachidonic acid from all the cells tested. In rat liver cells only tamoxifen stimulates basal prostaglandin I_2 _production and that induced by lactacystin and 12-O-tetradecanoyl-phorbol-13-acetate. LY117018, however, blocks the tamoxifen stimulated prostaglandin production. The stimulated prostaglandin I_2 _production is rapid and not affected either by preincubation of the cells with actinomycin or by incubation with the estrogen antagonist ICI-182,780.

**Conclusions:**

Tamoxifen and the raloxifene analog, LY117018, may prevent estrogen-independent as well as estrogen-dependent breast cancer by stimulating phospholipase activity and initiating arachidonic acid release. The release of arachidonic acid and/or molecular reactions that accompany that release may initiate pathways that prevent tumor growth. Oxygenation of the intracellularly released arachidonic acid and its metabolic products may mediate some of the pharmacological actions of tamoxifen and raloxifene.

## Background

The successful treatment and prevention of estrogen-dependent breast cancer in women by tamoxifen is attributed to its estrogen receptor (ER) occupancy [reviewed in [1,2]]. In the N-nitrosomethylurea (NMU) induced breast cancer model in rats, tumor growth is estrogen dependent and tamoxifen is considerably more effective than raloxifene [3]. In the dimethylbenzanthracene (DMBA)-induced model in rats, in which tumor growth is predominantly dependent on prolactin for growth, tamoxifen and raloxifene show effective anti-tumor action.

Tamoxifen and raloxifene have several properties in common; e.g. prevention of tumors in the DMBA induced rat mammary model, maintenance of bone density in the ovariectomized rat and reduction of low density lipoprotein cholesterol. The partial estrogen agonist activity of tamoxifen on uterine tissue, however, increases the risk of developing endometrial cancer. This does not appear to occur with raloxifene.

Tamoxifen stimulates arachidonic acid release from rat liver cells [4]. In this report, I have compared tamoxifen and the raloxifene analog LY117018 for effectiveness at releasing arachidonic acid (AA) from rat liver, rat glial, human colon carcinoma and human breast carcinoma cells and their effects on prostaglandin (PG) I_2 _production by the rat liver cells. Although both compounds release AA from these cells, LY117018 is less effective. Only tamoxifen stimulates both basal and PGI_2 _production induced by incubation of rat liver cells with lactacystin in the presence of 12-O-tetradecanoyl-phorbol-13-acetate (TPA). LY117018, however, inhibits the PGI_2 _production stimulated by tamoxifen.

The intracellular release of AA and/or the cellular reactions that accompany that release may initiate pathways that prevent tumor growth. The tissue specific effects of tamoxifen and LY117018 may be associated with the AA or with cyclooxygenase (COX) activity and/or one of the many bioactivities resulting from oxygenation and metabolism of the released AA.

## Methods

The C-9 rat liver and BT-20 human breast carcinoma cells were purchased from the American Type Culture Collection (Manassas, VA, USA) and maintained in MEM supplemented with 10% fetal bovine serum. The C-6 rat glial cell line was obtained from Dr. Elaine Y. Lai in the Department of Biology, Brandeis University and maintained in medium 199. The human colon carcinoma cells (HT-29) were obtained from Dr. Basil Rigas, American Health Foundation, Valhalla, NY and maintained in McCoy's medium. [^3^H]AA (91.8 Ci/mmol) was purchased from NEN Life Science Products, Inc. (Boston, MA, USA); ICI-182,780 from Tocris Cookson, Inc. (Ballwin, MO, USA); tamoxifen and 4-OH-tamoxifen were from Sigma Chemical Co. (St. Louis, MO, USA). LY117018 was obtained from Dr. David A. Cox, Eli Lilly and Co. (Indianapolis, IN, USA). Raloxifene was extracted from EVISTA^® ^tablets with dimethylsulfoxide.

Two days prior to experiments, the cells were treated with 0.25% trypsin-EDTA and, after addition of minimum essential medium (MEM), medium 199 or McCoy's medium containing 10% fetal bovine serum, the floating cells were seeded onto 35 mm culture dishes. The plating densities varied from 0.1 to 0.5 × 10^5 ^cells/35 mm dish. The freshly seeded cultures were incubated for 24-h to allow for cell attachment. After decantation of incubating media, 1.0 ml fresh media (MEM for the rat liver and BT-20 cells, medium 199 for the rat glial or McCoy's for the HT-29 cells respectively) containing 10% fetal bovine serum and [^3^H] AA (0.2 μCi/ml) was added and the cells incubated for 24-h. The cells were washed 4 times with MEM and incubated for various periods of time with 1.0 ml of MEM, medium 199 or McCoy's containing 1.0 mg bovine serum albumin (BSA)/ml and different concentrations of each test compound. The culture fluids were then decanted, centrifuged at 2000 × g for 10 min, and 200 μl of the supernate counted for radioactivity. Radioactivity recovered in the washes before the 6-h incubation was compared to input radioactivity to calculate the % radioactivity incorporated into the cells [5].

As measured by thin layer chromatography (TLC), of the [^3^H] released from radiolabelled methylcholanthrene transformed fibroblasts (about 20% after a 3-h stimulation by serum), 92% was AA, 4% was PGE_2_, 0.6% and 1% were PGF_2α _and phospholipids respectively [6]. When labelled to equilibrium, the [^3^H] AA had been incorporated into phosphatidylcholine (50%), phosphatidylethanolamine (36%), phodphatidylserine (9%) and triglycerides (10%) [7]. Such distribution varied among 12 cell lines [8]. The [^3^H] AA label released from human colorectal cancer cell lines (HCT116 and SW180) was AA as measured by TLC [9].

For PGI_2 _production by the rat liver cells, 1.0 ml of MEM supplemented with 10% fetal bovine serum, lacking [^3^H] AA, was added after the first 24-h incubation. The cells were incubated for another 24-h, washed three times with MEM, then incubated with lactacystin and TPA in the presence and absence of the test compounds in MEM/BSA for various periods of time. The culture fluids were decanted and analyzed for 6-keto-PGF_1α_, the stable hydrolytic product of PGI_2_, by radioimmunoassay [10]. At the sub-confluent cell densities used in these experiments (about 5 × 10^4 ^or less per dish) only the major COX product of rat liver cells (97% 6-keto-PGF_1α_) could be measured. The other two COX products, PGE_2 _(2%) and PGF_2α _(1%) could not be measured.

The release of [^3 ^H] AA is presented as a percentage of the radioactivity incorporated by the cells. Except for the time-course experiments, which used duplicate dishes (Fig. 5), three to six culture dishes were used for each experimental point. The data are expressed as mean values ± SEM. The data were evaluated statistically by the unpaired *Student's t-test*. A *P *value < 0.05 was considered significant.

## Results

### AA Release

**Figure 1:**
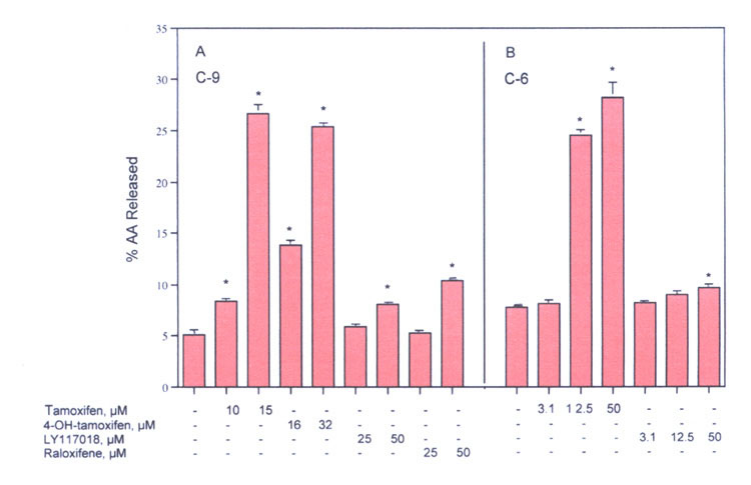
AA release from (A) rat liver cells (C-9) stimulated by tamoxifen, 4-OH-hydroxy-tamoxifen, LY117018 and raloxifene and (B) from rat glial cells incubated with tamoxifen and LY117018. The cells were incubated for 6-h. The analyses were performed with triplicate dishes. Each bar gives the mean value and the brackets give the SEM. * = statistically significant *vs *control. The data are representative of several independent experiments with similar results.

The release of AA after incubation of the rat liver cells with tamoxifen, 4-OH-tamoxifen, LY117018 and raloxifene for 6 h is shown in Fig. 1-A. Tamoxifen is the most effective: it releases AA at 10 μM. 4-OH-tamoxifen, an active metabolite of tamoxifen [2] is also effective. Raloxifene and LY117018 release AA from the rat liver cells, but are about 5 times less potent (at the doses tested) than tamoxifen. At μM concentrations, all induce apoptosis [11,12]. Tamoxifen, but not 4-OH-tamoxifen, has also been reported to induce apoptosis in p53(-) normal human mammary epithelial cells [13]. Yet, the metabolite binds to the ER with higher affinity than tamoxifen [14,15]. The release of AA by tamoxifen and LY117018 is not unique to the rat liver cells. Both release AA from rat glial cells (Fig. 1-B). Tamoxifen is more effective at releasing AA than is LY117018.

The highest level of [^3^H] AA released into the medium which contains BSA (1 mg/ml) would be 0.25 nM (the specific activity of the [^3^H] AA is 92 Ci/mmole). Since TLC or HPLC analyses were not done, the concentration of the total released AA could not be quantitatively estimated. However, even treatment of a colorectal cancer cell line with 200 μM AA for 48-h leads to apoptosis [9].

**Figure 2:**
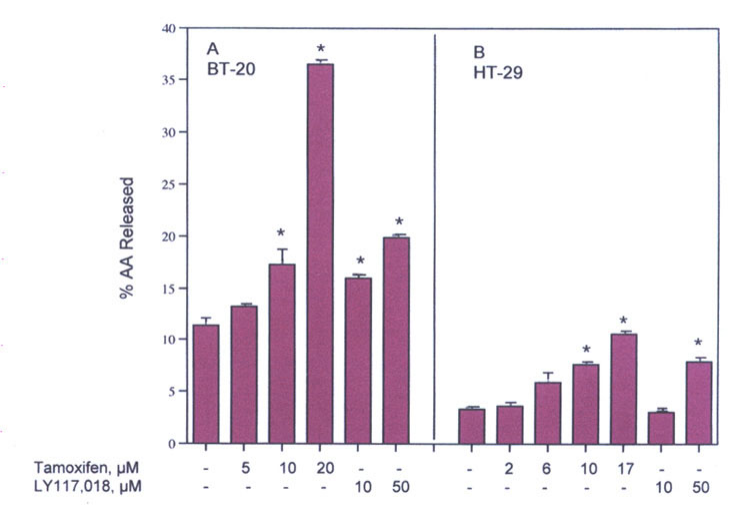
AA release from (A) human breast carcinoma cells (BT-20) and (B) human colon carcinoma cells (HT-29) by tamoxifen and LY117,018. The cells were incubated for 6-h. The analyses were performed with triplicate dishes. Each bar gives the mean values and the brackets give the SEM. * = statistically significant *vs *appropriate MEM/BSA control. The data are representative of several independent experiments.

The release of AA is neither species nor tissue specific. Both drugs also release AA from human breast carcinoma (BT-20) and human colon carcinoma (HT-29) cells (Fig. 2-A and 2-B). Again, LY117018 is less effective than tamoxifen.

### PGI_2 _production

**Figure 3:**
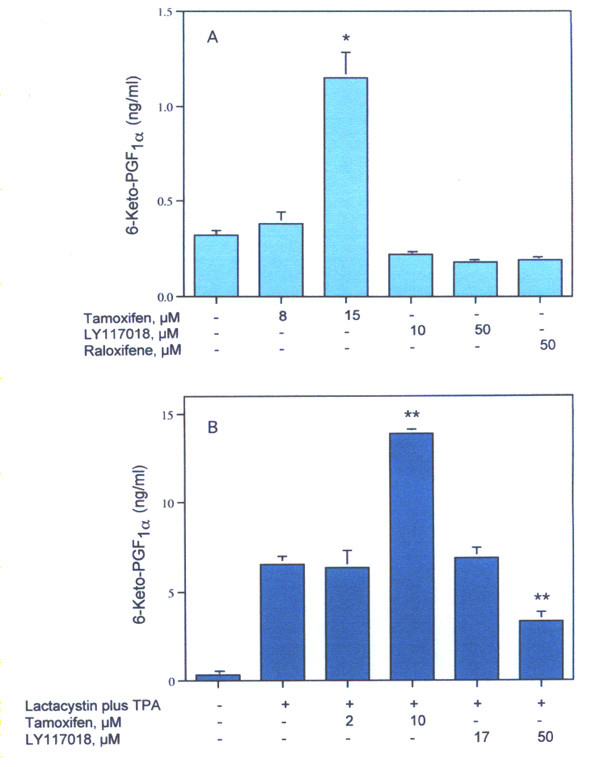
Effect of tamoxifen, LY117018 or raloxifene on (A) basal 6-keto-PGF_1α _production and (B) the effect of tamoxifen or LY117018 on 6-keto-PGF_1α _production induced by lactacystin in the presence of TPA. Rat liver cells were incubated for 6-h. The analyses were performed with triplicate or quadruplicate dishes. Each bar gives the mean value and the brackets the SEM. * = Statistically significant *vs *control stimulation. ** = Statistically significant *vs *lactacystin plus TPA control. The data are representative of several independent experiments with the same results.

Of the cultured cells studied, the COX mediated metabolic profile of AA has been described only in the rat liver cells. Thus, the effects of tamoxifen and LY117018 on PGI_2 _production by these cells were determined. Tamoxifen stimulates basal PGI_2 _production as well as that induced by lactacystin in the presence of TPA. LY117018 and raloxifene (Fig. 3-A) are ineffective in stimulating basal PGI_2 _production. LY117018, at a concentration that does not affect PGI_2 _production (50 μM), inhibits the PGI_2 _production stimulated by lactacystin in the presence of TPA (Fig. 3-B). The increased PGI_2 _production probably reflects some stimulation of both induced and basal COX activity. In mouse neuronal and embryonic rat mesencephalic cells, COX-2 is induced by lactacystin as measured by western and northern blot analyses [16].

**Figure 4:**
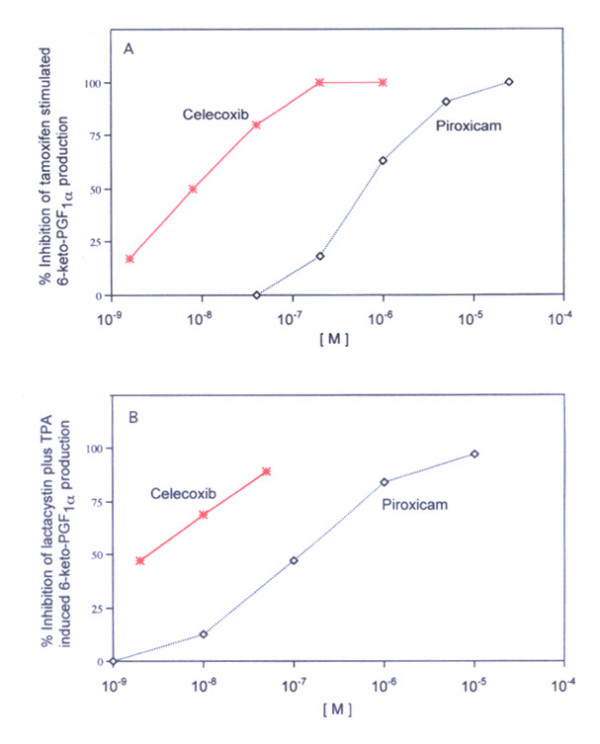
Inhibition of (A) tamoxifen (16 μM)-stimulated basal and (B) tamoxifen-stimulated induced 6-keto-PGF_1α _production (lactacystin in the presence of TPA) by celecoxib (*) and piroxicam (◊). Rat liver cells were incubated with either (A) tamoxifen (16 μM) or (B) lactacystin plus TPA in the presence of tamoxifen, for 6-h. The analyses were performed with triplicate dishes.

Identification of the induced and basal isoforms stimulated by tamoxifen in these rat liver cells was deduced from the relative effectiveness of inhibition of PGI_2 _production by celecoxib and piroxicam. Celecoxib is 7.5 times more effective than piroxicam as an inhibitor of COX-2, but 30,000 times less effective as an inhibitor of COX-1. Piroxicam is a selective inhibitor of COX-1 [17]. Celecoxib, the nonsteroidal anti-inflammatory drug (NSAID) that is a selective inhibitor of COX-2, inhibits both induced and basal COX activity 20–80 times more effectively than piroxicam (Fig. 4-A and 4B). Thus, based on inhibition by these two NSAIDs, COX-2 is the most likely isoform expressed both constitutively and after induction by lactacystin in the presence of TPA in rat liver cells. This conclusion was confirmed by western blot analyses (Levine L, Tashjian A, unpublished data).

### Is COX-2 production non-genomic and ER-independent?

**Figure 5:**
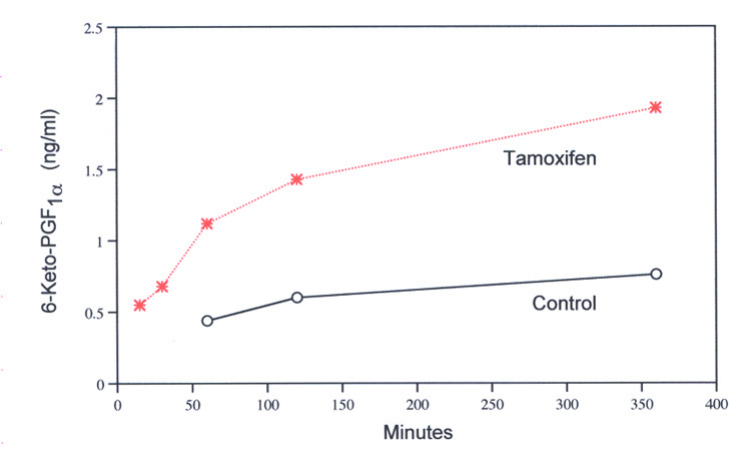
Time-course of 6-keto-PGF_1α _production by rat liver cells during incubation with 15 μM tamoxifen (γ) and MEM/BSA control (O). Analyses were performed with duplicate and triplicate dishes; mean values are shown.

**Figure 6:**
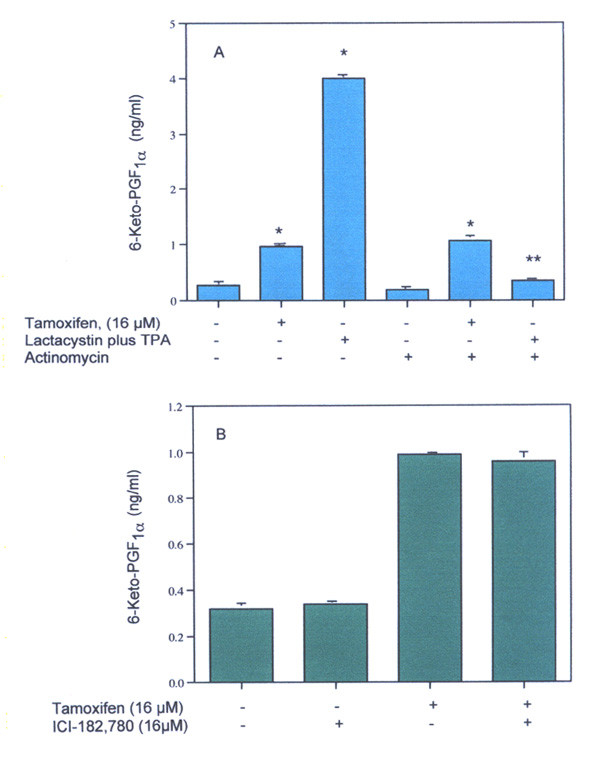
Effect of (A) actinomycin D on basal or induced (lactacystin plus TPA) 6-keto-PGF_1α _production stimulated by tamoxifen and (B) effect of ICI-182,780 on basal 6-keto-PGF_1α _production stimulated by tamoxifen. In (A), rat liver cells were preincubated with 1 μM actinomycin for 2-h and then incubated with tamoxifen (16 μM) or lactacystin plus TPA for 6-h. In (B), rat liver cells were incubated with tamoxifen (16 μM) and tamoxifen in the presence and absence of ICI-182,780 (16 μM) for 6-h. The analyses were performed with triplicate dishes. Each bar gives the mean value and the brackets give the SEM. Parts of the experiments were repeated several times with the same results. * Statistically significant *vs *control. ** Statistically significant *vs *lactacystin plus TPA.

The time-course of stimulation by tamoxifen (15 μM) of basal activity is shown in Fig. 5. Even after a 5-minute incubation (not shown in Fig. 5), stimulation by tamoxifen is significant statistically [0.08 ± 0.011 (n = 5) *vs *0.13 ± 0.011 (n = 5) ng 6-keto-PGF_1α _per ml in the absence and presence of tamoxifen, respectively]. Similar to AA release [4], stimulation of basal PGI_2 _production by tamoxifen is not affected by preincubation of the cells for 2-h with 1 μM actinomycin D. The induction obtained by incubation with lactacystin in the presence of TPA is inhibited (Fig. 6-A). Stimulation of basal PGI_2 _production by tamoxifen is unaffected by ICI-182,780 (Fig. 6-B). This estrogen antagonist, which binds to the ER with high affinity [18] did not affect the release of AA by tamoxifen [4] but does partially inhibit release of AA stimulated by 17β-estradiol, 22 (R)-cholesterol, indomethacin, *trans*-retinoic acid and the tyrosine analog of thiazolidinedione, GW7845 [19]. The rapidity of stimulation (5 min) and the lack of effect of actinomycin D or ICI-182,780 suggest that the stimulation of PGI_2 _production by tamoxifen is non-genomic and does not require ER occupancy.

**Figure 7:**
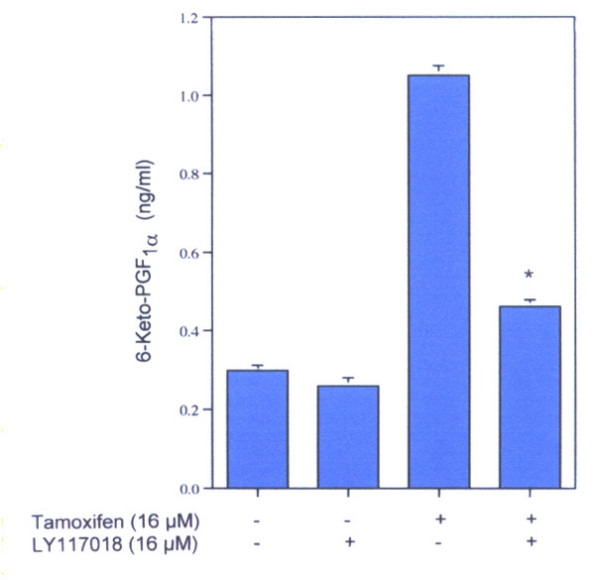
Effect of tamoxifen, LY117018 and tamoxifen plus LY118018 (all at 16 μM), on 6-keto-PGF_1α _production. The rat liver cells were incubated 6-h. The analyses were performed with quintuplicate dishes. Each bar gives the mean value and the brackets the SEM. * Statistically significant *vs *tamoxifen.

**Figure 8:**
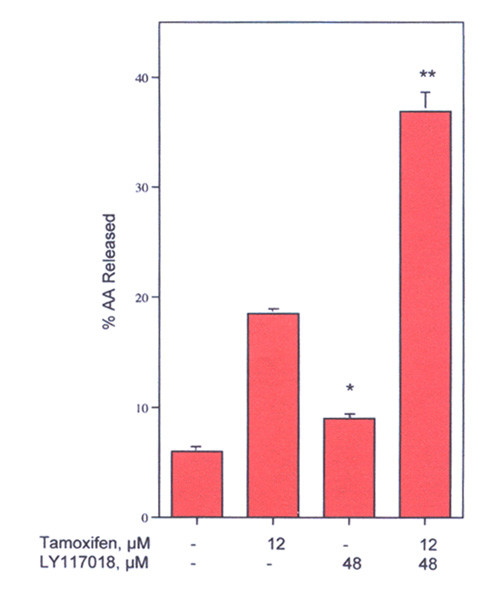
AA release from rat liver cells by tamoxifen (12 μM), LY117018 (48 μM) and tamoxifen in presence of LY117018. Cells were incubated for 6-h. The analyses were performed with triplicate dishes. Each bar gives the mean value and the brackets the SEM. * = Statistically significant *vs *MEM/BSA control. ** = Statistically significant *vs *tamoxifen.

LY117018 blocks PGI_2 _production stimulated by tamoxifen (Fig. 7). This blockade is unaffected by preincubation of the cells for 2-h with either actinomycin D or ICI-182,780 (data not shown). LY117018, however, does not inhibit the release of AA stimulated by tamoxifen; their combined effect is at least additive (Fig. 8).

## Discussion

These studies demonstrate that, at μM concentrations, tamoxifen and the raloxifene analog, LY117,018 stimulate the release of AA from cells in culture. At nM levels, the release is not observed. The effectiveness of these two compounds at prevention of estrogen-dependent breast cancer reflects competition for the ER [1,2]. In addition to occupancy of the ER, I am postulating that these drugs, at μM concentrations, may also prevent breast cancer of estrogen independent tumors. Consistent with this hypothesis are the findings that even at 100 μM concentrations, ethanol extracts of Femora ^® ^(letrozole) or Aramidex ^® ^(anastrozole) do not release AA from cells in culture (unpublished data). These two drugs inhibit estrogen synthesis by blocking aromatase enzymes and also prevent estrogen-dependent breast cancer [20]. In view of the stimulations of AA release by tamoxifen and LY117018 from human colon carcinoma cells (Fig. 2-A), the occurrence of colon cancer in women undergoing long term treatment with high levels of tamoxifen would be of interest.

A mechanism that most simply explains the release of AA by tamoxifen and LY117018 is the ability of such compounds to intercalate into cell membranes and affect phospholipase activities. The release of AA from endothelial cells by the Ca^2+ ^ionophore A-23187 reflects phospholipase activities. It is regulated by phosphorylation of the enzyme [21]. The enzymic properties of the altered membrane may impact signaling mechanisms e.g., pathways leading to apoptosis and COX induction. The intracellularly released AA also can serve as substrate for oxygenases. AA has intrinsic biologic activities that may also affect signaling pathways. Such changes would depend on the lipophilic properties of the compound and the composition of a particular membrane [22] and would vary from cell type to cell type, organelle to organelle and with the growth phase of the cell.

The AA release by tamoxifen and other reagents studied in my laboratory occurs with μM concentrations [4,5,19,23,24]. These experiments were carried out in the presence of BSA (1.0 mg/ml), and therefore do not differentiate between the protein-bound and free reagent. Thus, they are likely to be overestimated values. Nevertheless, the possibility that general necrotic cell death may cause AA release, must be considered. Tamoxifen, was found not to be toxic at concentrations of 10 to 20 μM for A549 human lung adenocarcinoma (ER-negative) cells [25]. Nor was 10 μM tamoxifen toxic when tested on rat glial cells and breast cancer MCF-7 cells [26]. Even when cell viability of three different breast cell lines (ER-positive MCF-7; ER-negative MDA-MB-239 and ER-negative BT-20 cells) was measured after incubation with 25 μM tamoxifen for 24-h, the loss in viability was due to apoptosis [12] and was not the result of necrotic cell death. Concentrations of tamoxifen used in this report are comparable to those found to induce apoptosis, not necrotic cell death. The median concentration of tamoxifen and its metabolites for clinical effectiveness in the treatment of breast cancer varies from 0.8 μM to 2.4 μM, depending on the age of the woman [27].

When measured after a 6-h incubation, 10 μM tamoxifen stimulates deesterification of membrane phospholipids as measured by extracellular AA release, (Fig. 1 and 2). The 6-h incubation may not be optimum for apoptosis to be observed, e.g. after a 6-h incubation of 5 μM tamoxifen with breast cancer cells, apoptosis was induced in about 10% of the cells compared to 8% in control cells [12]. After 24- and 48-h incubation, apoptosis in the tamoxifen treated cells increased to 40 and 70%, respectively. Apoptosis, induced by this membrane perturbation after 6-h incubation, increased 4 to 7 fold after longer incubation. It is apoptosis that may mediate the cancer preventative action of tamoxifen [11,12].

Some tissue specific effects of tamoxifen and raloxifene, e.g. on endometrial cells of the uterus, may be related to COX activity. At the low cell densities used in the present studies, the PGI_2 _produced by the rat liver cells can be quantitatively determined. The effects of tamoxifen and LY117018 on the COX activity of the rat liver cells may be similar to their effects on other cells that express COX. Tamoxifen and LY117018 affect COX activity differently; only tamoxifen stimulates PGI_2 _production. AA, which regulates the production of lipoxygenase, COX and cytochrome P-450 epoxygenase products could impact many of the pharmacological actions of these two selective estrogen receptor modulators. AA can be oxygenated by COX isoforms, lipoxygenases, and cytochrome P-450 epoxygenases and their products converted to the prostaglandins, leukotrienes, epoxyeicostetranoic acids [28] and AA to the oxidative stress-related isoprostanes [29]. In the COX pathway, enzymes convert PGH_2 _to the major physiologically active products, PGD_2_, PGE_2_, PGF_2α_, PGI_2 _and thromboxane A_2 _[28]. They, in turn, can be converted enzymatically and nonenzymatically to other biologically active compounds that possess different pharmacological properties. Often, only one major product is synthesized by the cell [8]. Thus, tissues can be affected differently by changes in COX activity. AA or its metabolites, would have different biological effects depending upon the genetic capabilities of the individual cell being affected. In addition to the spectrum of activities of the prostanoids, oxygenation of AA by 5-, 12- and 15-lipoxygenase and epoxygenases yield other products located in different cells and tissues [30,31].

## Competing interests

None declared.

## Author's contributions

L.L. is the sole author and all of the experiments, with the exception of the western blots referred to as Levine L and Tashjian A (unpublished data), were conducted by L.L.

## Pre-publication history

The pre-publication history for this paper can be accessed here:
